# Testicular cancer: Current management and controversial issues

**DOI:** 10.4103/0970-1591.60447

**Published:** 2010

**Authors:** Hemant Tongaonkar

**Affiliations:** Department of Urologic Oncology, Tata Memorial Hospital, Dr. E. Borges Road, Parel, Mumbai, India. E-mail: hemarmi@vsnl.net

Testicular cancers form 1% of all malignancies in men in India. Although uncommon, they affect men in the prime of their lives, being the most common solid tumor in young adults; with the potential to adversely impact their lives – both in terms of longevity and quality. It is a model for multidisciplinary management of cancer, with appropriate integration of surgery, chemotherapy and radiation therapy in the treatment protocols leading to high cure rates. The introduction of cisplatin-based chemotherapy has revolutionized the management of this cancer, with considerable improvement in response rates and excellent outcome reported even in the presence of metastatic disease.

Considerable effort has been put in to identify the clinicopathological and other parameters associated with the presence of occult metastases and selecting patients for additional therapy in patients with early stage testicular cancer. Consequently, the optimum individualized treatment for these patients is yet unclear. Long term results of randomized studies in terms of cancer-related outcome as well as morbidity, probably, will shed some light on this controversial issue.

With the achievement of high cure rates with appropriate use of cisplatin-based chemotherapy in patients with metastatic disease, the emphasis has now shifted to reducing morbidity of treatment and maintaining good quality of life in suitable patients without compromising chances of cure. With the incorporation of tumor markers in the staging system, these patients can now be sub stratified into different prognostic groups. The good prognosis patients have an excellent survival rate with the currently available chemotherapy protocols developed through well- designed cooperative group trials and the focus is on minimizing early and late morbidity without compromising cure. On the contrary, patients with poor prognostic features have a dismal survival with the standards treatment strategies and need more aggressive therapy delivered by an experienced multidisciplinary team in the hope of optimizing outcome. However, despite this aggressive approach, 20–30% of patients treated for metastatic disease currently die of the disease. This emphasizes the need for well-conceived randomized trials to define newer therapies for this group of patients.

Despite effective chemotherapy, a significant number of patients with initial metastatic disease have persistent residual masses after chemotherapy. Although complete removal of all post-chemotherapy residual masses remains the standard of care and allows improved prognostication of the long term functional and oncological outcome, the indications, extent and benefit of this extirpative surgery remains controversial. It is necessary to identify better predictive criteria that could accurately assess the histology of these residual masses.

The risk of overtreatment, long term sequelae and morbidity, which was till recently not well appreciated, and the ability to achieve equivalent results with more than one competing modalities has raised many controversies in the management of this cancer.

All the contributing authors are experts in the field of management of testicular cancer and have done an excellent job in reviewing the current state-of-the-art and making balanced recommendations based on the best available evidence. They have tackled the contentious and controversial issues in a very objective manner and I am sure their contribution will help urologists and uro-oncologists treating testicular cancer in making proper management decisions. I am extremely grateful to them for having spared invaluable time from their busy schedules to contribute to this symposium.

